# Early Secreted Antigenic Target of 6-kDa of *Mycobacterium tuberculosis* Stimulates IL-6 Production by Macrophages through Activation of STAT3

**DOI:** 10.1038/srep40984

**Published:** 2017-01-20

**Authors:** Bock-Gie Jung, Xisheng Wang, Na Yi, Justin Ma, Joanne Turner, Buka Samten

**Affiliations:** 1 Department of Pulmonary Immunology, University of Texas Health Science Center at Tyler, Texas 75708, USA; 2 Department of Microbial Infection and Immunity and Center for Microbial Interface Biology, the Ohio State University, Columbus, OH 43210, USA.

## Abstract

As early secreted antigenic target of 6 kDa (ESAT-6) of *Mycobacterium tuberculosis (Mtb*) is an essential virulence factor and macrophages are critical for tuberculosis infection and immunity, we studied ESAT-6 stimulated IL-6 production by macrophages. ESAT-6 stimulated significantly higher IL-6 secretion by murine bone marrow derived macrophages (BMDM) compared to culture filtrate protein 10 kDa (CFP10) and antigen 85A. Polymyxin B, an LPS blocker, did not affect ESAT-6 stimulated macrophage IL-6 production. ESAT-6 but not Pam3CSK4 induced IL-6 by TLR2 knockout BMDM. ESAT-6 induced phosphorylation and DNA binding of STAT3 and this was blocked by STAT3 inhibitors but not by rapamycin. STAT3 inhibitors suppressed ESAT-6-induced IL-6 transcription and secretion without affecting cell viability. This was confirmed by silencing STAT3 in macrophages. Blocking neither IL-6Rα/IL-6 nor IL-10 affected ESAT-6-induced STAT3 activation and IL-6 production. Infection of BMDM and human macrophages with *Mtb* with *esat-6* deletion induced diminished STAT3 activation and reduced IL-6 production compared to wild type and *esat-6* complemented *Mtb* strains. Administration of ESAT-6 but not CFP10 induced STAT3 phosphorylation and IL-6 expression in the mouse lungs, consistent with expression of ESAT-6, IL-6 and phosphorylated-STAT3 in *Mtb*-infected mouse lungs. We conclude that ESAT-6 stimulates macrophage IL-6 production through STAT3 activation.

*Mycobacterium tuberculosis (Mtb*), the causative agent of tuberculosis (TB), has become one of the deadliest pathogens in human history. This is mainly due to lack of effective TB vaccine, widespread human immunodeficiency virus (HIV) co-infection in TB endemic countries and development of drug resistance by *Mtb*. Furthermore, development of strategies by *Mtb* to evade host immunity to persist and cause disease^1^ is also a critical contributing factor. Therefore, deciphering the interaction between immune cells and the virulence factors of *Mtb* is the first step toward development of an effective vaccine and finding novel therapeutics for TB control.

Recent studies have established that secretion of early secreted antigenic target 6-kDa (ESAT-6) and its molecular partner, culture filtrate protein 10 kDa (CFP10), through ESAT-6 (ESX)-1 secretion system, is essential for *Mtb* pathogenesis^2^, but how ESAT-6 and CFP10 cause pathology remains obscure. Although tissues destruction due to cell damage is potentially one of the mechanisms by which ESAT-6 causes pathology because ESAT-6 causes lysis of alveolar epithelial cells and macrophages^3^,^4^ and apoptosis^5^ and necrosis of macrophages^6^, our previous studies suggest that ESAT-6 mediated regulation of host immune responses may play a role in *Mtb* pathogenesis. ESAT-6 directly inhibits T cell IFN-γ production by activation of p38 MAPK^7^,^8^ and indirectly through reprogramming of antigen presenting cells to produce less IL-12p70, an essential IFN-γ stimulating cytokine, and more IL-23, IL-1β and probably IL-6, the Th17 supporting cytokines^8^,^9^. We also showed that ESAT-6 induces IL-8 production by lung epithelial cells to promote granuloma formation^10^. These findings suggest that ESAT-6 has the potential to manipulate host immunity during *Mtb* infection.

Alveolar macrophages play key roles in TB infection by acting both as an intracellular niche for and as a first line defense against *Mtb* infection by phagocytosis of *Mtb*, production of cytokines and presenting *Mtb* antigens to initiate and regulate *Mtb* specific adaptive immunity^11^,^12^,^13^. It was suggested that virulent *Mtb* manipulates host immune responses through macrophages^14^.

Interleukin (IL)-6 is a multifunctional cytokine produced by various cell types including macrophages, to regulate normal physiological processes^15^, such as hematopoiesis, acute phase inflammatory response and immune responses. However, dysregulation of IL-6 production is associated with various diseases, such as cancer^16^ and HIV infection^17^. Macrophages from TB patients produce higher levels of IL-6 than those from healthy subjects^18^ and elevated circulating IL-6 levels were found in the patients with far-advanced pulmonary TB lesions^19^. Indeed, initial identification of IL-6 was accomplished by investigating IL-6 purified from the culture supernatants of purified protein derivative stimulated pleural effusion cells from patients with tuberculous pleurisy^20^. Furthermore, infection of macrophages by mycobacterial species induces IL-6 which is responsible for suppression of Th1 responses^21^ and suppression of *Mtb* infected and non-infected bystander macrophage responses to IFN-γ^22^. IL-6 also inhibits IFN-γ induced autophagy in *Mtb* infected macrophages^23^. Thus, these findings clearly indicate that virulent *Mtb* may upregulate IL-6 production, especially by macrophages, to regulate host immunity and susceptibility to TB. Therefore, we examined whether ESAT-6 induces IL-6 production by macrophages and the role of signal transducer and activator of transcription (STAT)3 in this process. We demonstrated that ESAT-6 induces IL-6 production by macrophages through activation of STAT3.

## Results

### ESAT-6 stimulates IL-6 production by macrophages

**Figure 1:**
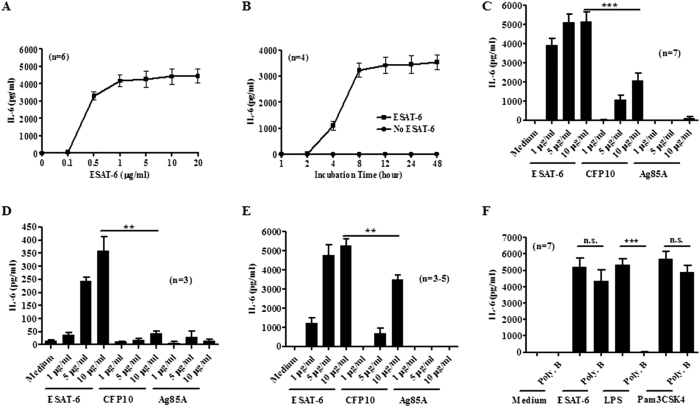
ESAT-6 stimulates IL-6 production by macrophages. Mouse BMDMs (**A**) were treated with or without ESAT-6 as indicated and IL-6 levels in the culture supernatants were determined after 24 h incubation. (**B**) BMDMs were incubated with or without ESAT-6 at 1 μg/ml for various times and IL-6 levels were determined. BMDMs (**C**), mouse alveolar macrophages (**D**) and RAW 264.7 cells (**E**) were incubated with indicated concentrations of proteins and IL-6 levels after 24 h incubation were determined. (**F**) BMDMs treated with or without 10 μg/ml polymyxin B (Poly. B) for 1 h prior to incubation with medium alone, ESAT-6 (1 μg/ml), LPS (0.1 μg/ml) or Pam3CSK4 (0.1 μg/ml) for 24 h and IL-6 levels were determined. For all panels, data are expressed as means ± SEM, *P < 0.05, **P < 0.01 or ***P < 0.001. n.s., not significant.

Although IL-6 is required for protective immunity, elevated IL-6 production correlates with disease severity of TB patients^24^. Excess IL-6 production may lead to suppressed Th1 responses^21^ and failed IFN-γ driven anti-*Mtb* responses of macrophages^22^. Therefore, we determined whether ESAT-6 stimulates IL-6 production by macrophages. ESAT-6 induced IL-6 production by BMDMs in a dose dependent manner (Fig. 1A), started to induce IL-6 at as low as 0.5 μg/ml (80 nM) and peaked at 1 μg/ml (160 nM) after 24 h stimulation. As for the temporal effect, we incubated BMDMs with 1 μg/ml ESAT-6 for different time points. ESAT-6 induced production of IL-6 as early as 1 h after stimulation from nondetectable level without ESAT-6 to 9.3 ± 1.3 pg/ml, at 2 h from 1.5 ± 1.5 pg/ml without ESAT-6 to 19.5 ± 6.8 pg/ml, peaked at 8 h and plateaued thereafter (Fig. 1B). As controls, we used CFP10 and Ag85A prepared as ESAT-6 in our laboratory. Although CFP10 at 5 and 10 μg/ml induced IL-6 production by macrophages, which are significantly less than that stimulated by ESAT-6 at same concentrations, and Ag85A did not stimulate any IL-6 production by macrophages at all three concentrations (Fig. 1C). We also checked whether ESAT-6 induces IL-6 production by primary alveolar macrophages and alveolar macrophage like cell line, RAW264.7 cells. Though IL-6 levels were lower than that by BMDMs, ESAT-6 stimulated significantly elevated IL-6 production by alveolar macrophages in a dose dependent manner compared to the cells with CPF10, Ag85A or with medium alone (Fig. 1D). Similarly, ESAT-6 also stimulated significant amount of IL-6 by RAW 264.7 cells (Fig. 1E). Although CFP10 at 10 μg/ml induced IL-6 production by RAW264.7 cells, which was significantly less than that by same concentration of ESAT-6 and Ag85A did not induce IL-6 production by these cell types (Fig. 1D and E). These data indicate that ESAT-6 stimulates IL-6 production by macrophages including alveolar macrophages.

To test whether the trace amounts of LPS in ESAT-6 was the cause for IL-6 induction by macrophages, we used polymyxin B, a neutralizer of LPS^25^. Polymyxin B at 10 μg/ml did not affect 1 μg/ml ESAT-6 stimulated macrophage IL-6 production and completely blocked that by 100 ng/ml LPS, which is over 1,000 fold higher in LPS content than that in ESAT-6 at 1 μg/ml. The effect of polymyxin B was specific, as it did not affect IL-6 stimulation by TLR2 agonist Pam3CSK4 (Fig. 1F). These results suggest that ESAT-6 stimulated IL-6 production by macrophages is due less likely to LPS contamination.

### ESAT-6 activates STAT3 in macrophages

**Figure 2:**
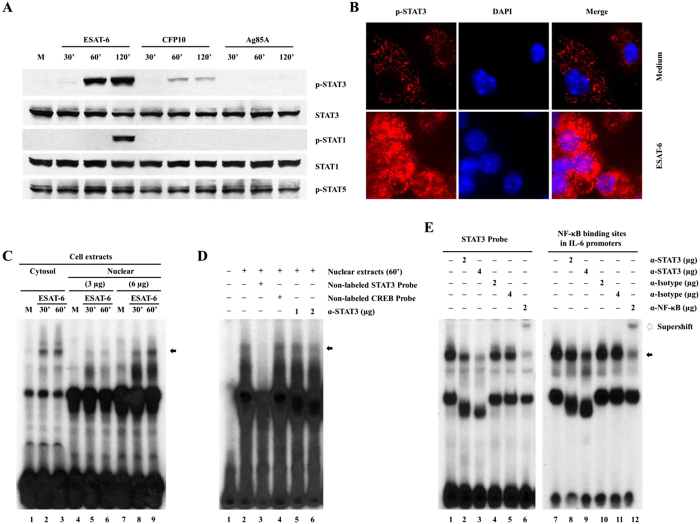
ESAT-6 activates STAT3 in macrophages. (**A**) BMDMs were incubated with 1 μg/ml ESAT-6, CFP10 or Ag85A for different periods and the expression of the proteins was determined by Western blotting. One representative result of five independent experiments is shown. (**B**) BMDMs on cover slides were incubated with 1 μg/ml ESAT-6 for 60 min, fixed and stained for phospho-STAT3 and the cell nucleus was visualized with DAPI. One representative result from five different experiments is shown. (**C**) BMDMs were incubated with 1 μg/ml ESAT-6 for different time points and the DNA binding activities in nuclear and cytosolic protein extracts were evaluated by EMSA using labeled STAT3-binding site as probe. The arrow shows the specific protein-DNA complex. (**D**) STAT3 specific DNA binding was tested by EMSA as in (**C**) using nuclear extracts of BMDMs with ESAT-6 for 60 min. Non-labeled STAT3 (lane 3) and CREB binding site (lane 4) were used as cold competitors. STAT3 mAb were used at two different concentrations (lanes 5 and 6). (**E**) EMSA was performed as in (**C**), with or without different Abs as indicated using total protein extracts of BMDMs treated with ESAT-6 for 60 min and labeled STAT3-binding site and IL-6 proximal promoter as probes. For panels C, D, and E, one representative result from three independent experiments is shown.

To elucidate the signaling pathways, we focused on STAT3 as it plays critical role in IL-6 production in cancer and infectious diseases^26^,^27^ and STAT3 and its regulator suppressor of cytokine signaling (SOCS)3 are critical in TB^28^,^29^. To this end, we evaluated phosphorylation of STAT3 in macrophages stimulated with ESAT-6. ESAT-6 at 1 μg/ml induced STAT3 phosphorylation at Tyr-705 in a time-dependent manner (Fig. 2A) compared to the cells without ESAT-6. In contrast, 1 μg/ml CFP10 induced STAT3 phosphorylation which was significantly less than that induced by ESAT-6. Ag85A did not induce STAT3 phosphorylation at all three time points tested. We examined the phosphorylation of STAT3 at Ser-727 and acetylation, but we could not detect any such modifications of STAT3 in ESAT-6 stimulated macrophages (results not shown). We also determined STAT1 phosphorylation in this process. ESAT-6 induced STAT1 phosphorylation only at 120 min, which was later than ESAT-6-induced STAT3 phosphorylation and was probably due to a secondary effect of ESAT-6-induced type I IFNs by macrophages^30^. CFP10 and Ag85A did not induce STAT1 phosphorylation at any of the time points. None of these proteins affected phosphorylation of STAT-5 in macrophages. These were not due to differential protein loading as comparable total STAT3 and STAT1 were detected across the lanes in the same membrane after stripping.

To demonstrate ESAT-6 activation of STAT3 in macrophages by an alternative method, we performed immunofluorescence staining and confocal microscopy analysis. Stimulation of macrophages with ESAT-6 at 1 μg/ml for 60 min induced STAT3 phosphorylation. The phosphorylated STAT3 was located both in the nucleus and cytoplasm though with dominance in the cytoplasm (Fig. 2B), consistent with a previous report that activated STAT3 is not only localized in the nucleus but also in the cytoplasm, probably in mitochondria^31^.

To test the functional significance of ESAT-6 activated STAT3, we examined DNA binding activity in the protein extracts of ESAT-6-treated macrophages by EMSA using a labeled STAT3 binding site as probe. ESAT-6 induced STAT3 binding activities in both the cytoplasmic and nuclear protein extracts of macrophages in a time dependent manner compared to the cells without ESAT-6 (Fig. 2C), consistent with our confocal microscopy data, indicating activation of STAT3 in both cell cytoplasm and nuclear. The STAT3 binding activity is specific since the presence of excess unlabeled STAT3 but not CREB binding site blocked the formation of DNA-protein complex and the presence of anti-STAT3 antibody also blocked this interaction in a concentration dependent manner (Fig. 2D, lanes 5 and 6).

To determine the identities of the STAT3-binding proteins induced by ESAT-6, we performed EMSA supershift assays by incubating the labeled STAT-3 binding site or the proximal promoter of IL-6 as probes with total protin extracts of macrophages treated with ESAT-6 for 60 min followed by incubation with Abs. ESAT-6 induced a strong high molecular weight protein-DNA complex with either STAT3 binding site or the proximal promoter of IL-6 (Fig. 2E, lane 1 and 7 for STAT3 binding site and IL-6 promoter, respectively), and this was disrupted by anti-STAT3 Ab dose dependently (Fig. 2E, lanes 2 and 3 for STAT3 binding site and lanes 8 and 9 for IL-6 promoter) and supershifted by anti-NF-κBp65 (Fig. 2E, lanes 6 and 12) but not affected by control IgG (Fig. 2E, lanes 4 and 5 and, 10 and 11). These results demonstrated that ESAT-6 activates both STAT3 and NF-κB in macrophages and both of these may participate IL-6 gene transcription through the proximal promoter of IL-6 as a complex. Taken together, the results demonstrated that ESAT-6 indeed stimulates activation of STAT3 and may induce transcription of IL-6.

### Pharmacological inhibition of STAT3 blocks ESAT-6-induced IL-6 production

**Figure 3:**
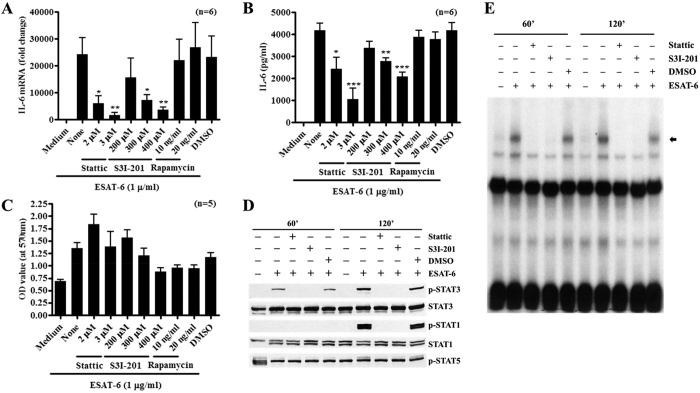
Inhibition of STAT3 abrogates ESAT-6 induced macrophage IL-6 expression and STAT3 activation. BMDMs were incubated with different inhibitors as indicated for one hour prior to incubation with ESAT-6. IL-6 mRNA expression was determined by qPCR after 4 h incubation (**A**), IL-6 was determined in the culture supernatants (**B**) and the viability of the cells was determined by MTT assay (**C**) after 24 h incubation. BMDMs were treated with STAT3 inhibitors Static (3 μM) and S31-201 (400 μM) or DMSO for one h before incubation with 1 μg/ml ESAT-6 for 60 and 120 min. The activation of STAT proteins was determined by Western blotting (**D**) and the DNA binding was determined by EMSA (**E**) in the total cell protein extracts. For panels A–C, data expressed as Mean ± SEM, *P < 0.05, **P < 0.01 or ***P < 0.001. For panels D and E, one representative result of 4 independent experiments is shown.

To verify the role of STAT3 in ESAT-6-induced IL-6 production by macrophages, we applied Stattic and S3I-201, the chemical inhibitors of STAT3. Pretreatment of macrophages with Stattic or S3I-201, reduced ESAT-6 induced IL-6 mRNA expression (Fig. 3A) and IL-6 secretion (Fig. 3B) by macrophages in a dose dependent manner compared to the cells without inhibitors or with DMSO, vehicle control. In contrast, pretreatment of macrophages with mTOR inhibitor, rapamycin, did not affect ESAT-6 stimulated IL-6 production, implying a specific effect of STAT3 inhibitors. To verify this was not due to cytotoxicity of the chemicals, we evaluated cell viability using MTT assay. ESAT-6 induced elevated metabolic activity of the macrophages compared to the cells without ESAT-6 and STAT3 inhibitors did not affect cell viability except for S3I-201at 400 μM. Rapamycin at both concentrations slightly reduced the cell viability compared to the cells with ESAT-6 alone (Fig. 3C). These together argue against the cytotoxicity as the cause for the reduced ESAT-6 induced IL-6 production by macrophages in the presence of STAT3 inhibitors. To further examine this in detail, we tested the effect of STAT3 inhibitors on ESAT-6 induced activation of STAT3. Again, ESAT-6 induced phosphorylation of STAT3 at 60 and 120 min and phosphorylation of STAT1 at 120 min compared to the cells without ESAT-6 (Fig. 3D), and this was completely blocked by STAT3 inhibitors but not DMSO. Consistent with this, ESAT-6 induced the high molecular weight protein-DNA complex at both 60 and 120 min after stimulation and this was completely disrupted by STAT3 inhibitors but not by DMSO (Fig. 3E), implying reduced phosphorylation of STAT3 by its inhibitors in macrophages stimulated with ESAT-6. These results clearly showed that ESAT-6 induces expression and secretion of IL-6 by macrophages through activation of STAT3.

### Silencing STAT3 in macrophages leads to reduced IL-6 stimulation by ESAT-6

**Figure 4:**
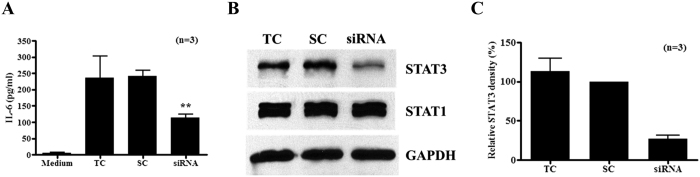
Silencing STAT3 in macrophages reduces ESAT-6 stimulated IL-6 production. BMDMs were transfected with transfection reagent only (TC), scrambled (SC) and STAT3 siRNA for 48 h. The cells were stimulated with ESAT-6 at 1 μg/ml for 24 h and IL-6 levels were determined in the culture supernatants (**A**). The silencing efficiency was determined by Western blotting for STAT3 in the cellular lysates after 48 h transfection. As controls, STAT1 and GAPDH were blotted in the same membrane after stripping. One representative result of three experiments (**B**) is shown. The Means and SEM of relative densities of STAT3 bands were calculated using the density of STAT3 in cells with scrambled siRNA after normalized by densities of respective STAT1 bands from three experiments (**C**).

Although chemical inhibitors are convenient and critical tools for understanding the specific functions of proteins of interest, they may have off-target effects. To confirm the role of STAT3 in ESAT-6 induced IL-6 production by macrophages, we applied RNAi silencing technology. Although overall IL-6 levels were lower than the previous experiments probably because of transfection reagent treatment and extended incubation times, the mock or scrambled siRNA transfected BMDM responded with elevated IL-6 production to ESAT-6 compared to the cells without ESAT-6, and this was reduced significantly by STAT3 siRNA transfection (Fig. 4A) and the silencing efficiency was confirmed to be over 80% by Western blotting followed by densitometry analysis (Fig. 4B and C). The results from both biochemical inhibitors and genetic silencing studies support a role for STAT3 in ESAT-6 stimulated IL-6 production by macrophages.

### Neither TLR-2 nor autocrine effect of IL-6 or IL-10 is involved in ESAT-6-induced STAT3 activation and IL-6 production by macrophages

**Figure 5:**
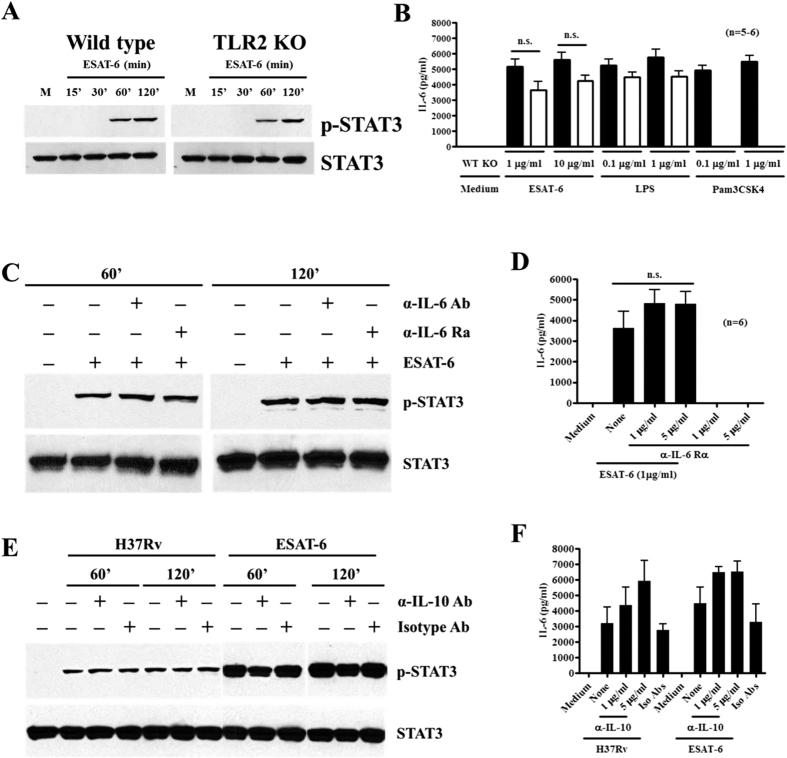
ESAT-6 stimulates STAT3 phosphorylation and IL-6 production by macrophages independent of TLR2 or autocrine effects of IL-6 and IL-10. BMDMs from three wild-type or TLR2−/− mice were incubated with ESAT-6 for different time points and the phosphorylation of STAT3 was determined by Western blotting followed by blotting for total STAT3 in the same membrane after stripping (**A**). BMDMs from 5 wild-type (filled bars) or 6 TLR2−/− (open bars) mice were incubated with medium alone or indicated concentrations of ESAT-6, LPS or Pam3CSK4 and IL-6 levels were determined 24 h later (**B**). Data are expressed as means ± SEM. BMDMs from wild-type mice were incubated with either anti-IL-6 or anti-IL-6R for one hour prior to addition of ESAT-6 at 1 ug/ml for different time points. The phosphorylation of STAT3 and total STAT3 (**C**) was determine as in (**A)** and IL-6 production (**D**) was determined as in (**B)**. BMDMs were incubated with anti-IL-10 at 5 μg/ml for one hour prior to stimulation with ESAT-6 or infection with H37Rv. Activation of STAT3 (**E**) and production of IL-6 (**F**) were determined as in (**A** and **B**), respectively. One representative result of three independent experiments is shown for (**A**,**C** and **E**). The data are expressed as means ± SEM for (**B**,**D** and **F)**.

It was reported that ESAT-6 regulates macrophage cytokine production through TLR2^32^,^33^,^34^. Therefore, we tested this using BMDMs from both wild-type and TLR2KO mice. ESAT-6 at both concentrations stimulated IL-6 production by wild-type macrophages at comparable levels to that induced by both Pam3CSK4 and LPS, TLR2 and TLR4 agonists, respectively. With TLR2KO macrophages, ESAT-6 stimulated IL-6 production to the same extent as LPS though overall IL-6 responses were slightly lower than wild-type macrophages, and as expected Pam3CSK4 at both concentrations did not stimulate any detectable levels of IL-6, (Fig. 5B). TLR2KO macrophages responded with phosphorylation of STAT3 at the same level as wild type macrophages did upon ESAT-6 stimulation (Fig. 5A). These results clearly argue against the role of TLR2 in ESAT-6-induced STAT3 phosphorylation and IL-6 production by macrophages. Since cytokines, such as IL-6 and IL-10 are major activators of STAT3^35^ and indeed IL-10 was shown to be responsible for STAT3 phosphorylation in macrophages infected with *Mtb*^36^, we examined the effect of these two cytokines on ESAT-6 and H37Rv induced phosphorylation of STAT3 and IL-6 production by macrophages. Blocking IL-6 or IL-6R did not affect ESAT-6 induced activation of STAT3 (Fig. 5C) and production of IL-6 by macrophages (Fig. 5D). Neutralizing IL-10 did not affect the level of STAT3 phosphorylation in macrophages stimulated by ESAT-6 or infected with H37Rv (Fig. 5E). Neutralizing IL-10 resulted in elevated IL-6 production by macrophages in response to either ESAT-6 or H37Rv (Fig. 5F), consistent with previous report^37^ and confirming the neutralizing activity of anti-IL-10 used. These results together argue against the potential autocrine or secondary effects of IL-6 and IL-10 in STAT3 activation and IL-6 production by macrophages in response to ESAT-6 stimulation and *Mtb* infection.

### Mtb lacking ESAT-6 induces less IL-6 production and weak STAT3 activation in macrophages

**Figure 6:**
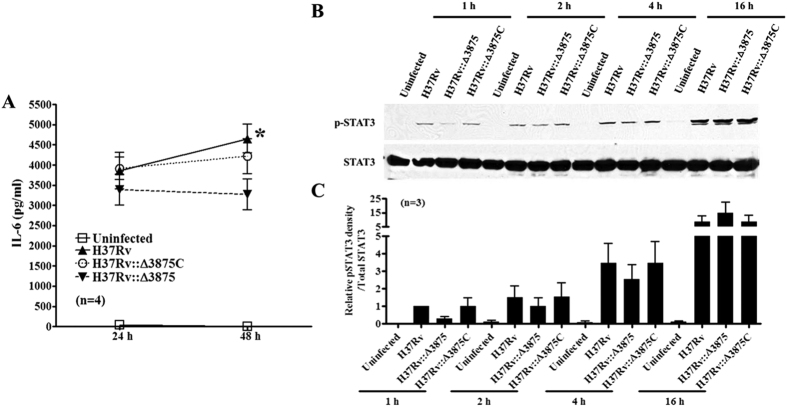
*Mtb* with *esat-6* deletion is less potent in STAT3 activation and IL-6 production by macrophages. BMDMs were infected with *Mtb* wild type strain (H37Rv), its *esat-6* deletion mutant (H37Rv::Δ3875) or *esat-6* complemented strain (H37Rv::Δ3875C) at 10 multiplicity of infection and IL-6 levels in the culture supernatants were determined by ELISA (**A**) and expressed as means ± SEM. Some cells were collected at 1, 2, 4 and 16 h post infection and phospho-STAT3 was determined by Western blotting followed by blotting for total STAT3 after stripping (**B**). One representative result of 3 independent experiments is shown (**B**). Means and SEM of relative densities of phospho-STAT3 were calculated using the densities of phospho-STAT3 with H37Rv at 1 h as 1 and after normalized by the respective densities of total STAT3 from three different experiments (**C**).

To determine whether our findings from recombinant ESAT-6 studies are also applicable to live *Mtb* infection, we infected BMDMs with *Mtb* strain H37Rv, its *esat-6* deletion mutant H37Rv::Δ3875 or *esat-6* complemented strain H37Rv::Δ3875C^38^ and determined the activation of STAT3 and production of IL-6. Although all three strains induced IL-6 production by BMDMs compared to uninfected control cells (medium), H37Rv::Δ3875 induced less IL-6 production by macrophages at 24 h and 48 h with significant reduction at 48 h post infection compared to H37Rv (Fig. 6A). In contrast, *esat-6* complemented strain restored the capacity of IL-6 stimulation by macrophages. Similarly, *esat-6* deletion mutant induced less STAT3 phosphorylation in macrophages than both wild type and *esat-6* complemented strains at 1 (70%), 2 (33.5%) and 4 h (26.2%) post infection (Fig. 6B and C). These indicate that ESAT-6 may contribute to STAT3 activation and IL-6 production by macrophages infected with *Mtb*.

### ESAT-6 stimulates activation of STAT3 and production of IL-6 by human macrophages

**Figure 7:**
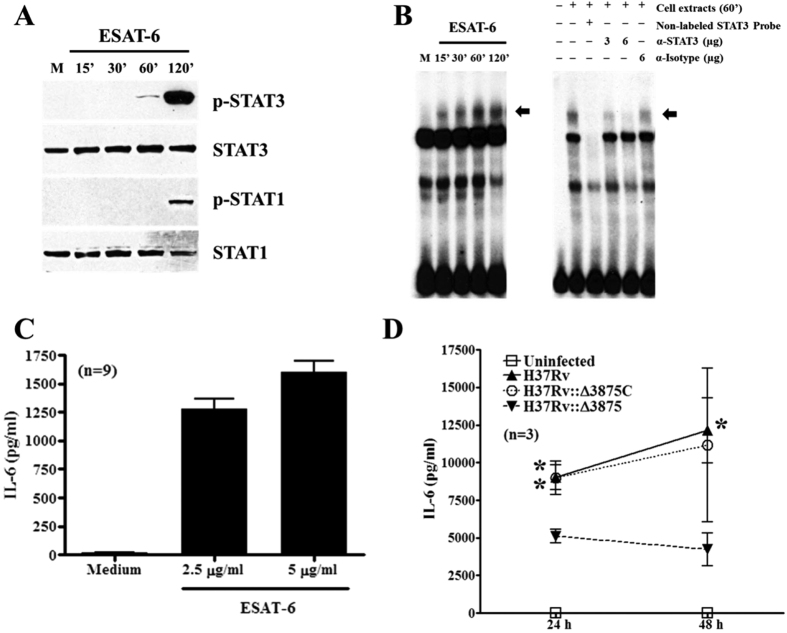
ESAT-6 stimulates STAT3 activation and IL-6 production by human macrophages. Human monocyte derived macrophages were incubated with 1 μg/ml ESAT-6 for different periods and phospho-STAT3 and phospho-STAT1 were determined by Western blotting followed by blotting for total STAT3 and STAT1 after stripping (**A**) in cellular protein extracts. The DNA binding activity in the cellular protein extracts was determined by EMSA using labeled STAT3 as probe. The specificity of binding and identity of STAT3 was confirmed by competition and supershift EMSA as indicated (**B**). The arrow indicates specific DNA-protein complex. Some cells were stimulated with ESAT-6 at indicated concentrations and IL-6 levels were determined in 24 h culture supernatants (**C**). Human macrophages were infected with different strains of *Mtb* as described in Fig. 6A and IL-6 levels were determined at 24 and 48 h later (**D**).

To confirm our findings from mouse macrophages, we performed experiments with human peripheral blood monocyte derived macrophages. The results show that ESAT-6 at 1 μg/ml induced phosphorylation of STAT3 and STAT1 with the same pattern as in murine BMDMs (Fig. 7A) and induced strong STAT3 binding activity and this was suppressed by anti-STAT-3 antibody but not by isotype control IgG (Fig. 7B). Consistent with this, ESAT-6 also induced strong IL-6 production by macrophages (Fig. 7C). Infection of human monocyte derived macrophages with wild-type and *esat-6* complemented strain of H37Rv induced strong IL-6 production compared with the cells with *esat-6* deletion mutant H37Rv::Δ3875 (Fig. 7D), further supporting the data from mouse macrophages.

### Infection of Mtb and administration of ESAT-6 induces phosphorylation of STAT3 and expression of IL-6 in mouse lungs

**Figure 8:**
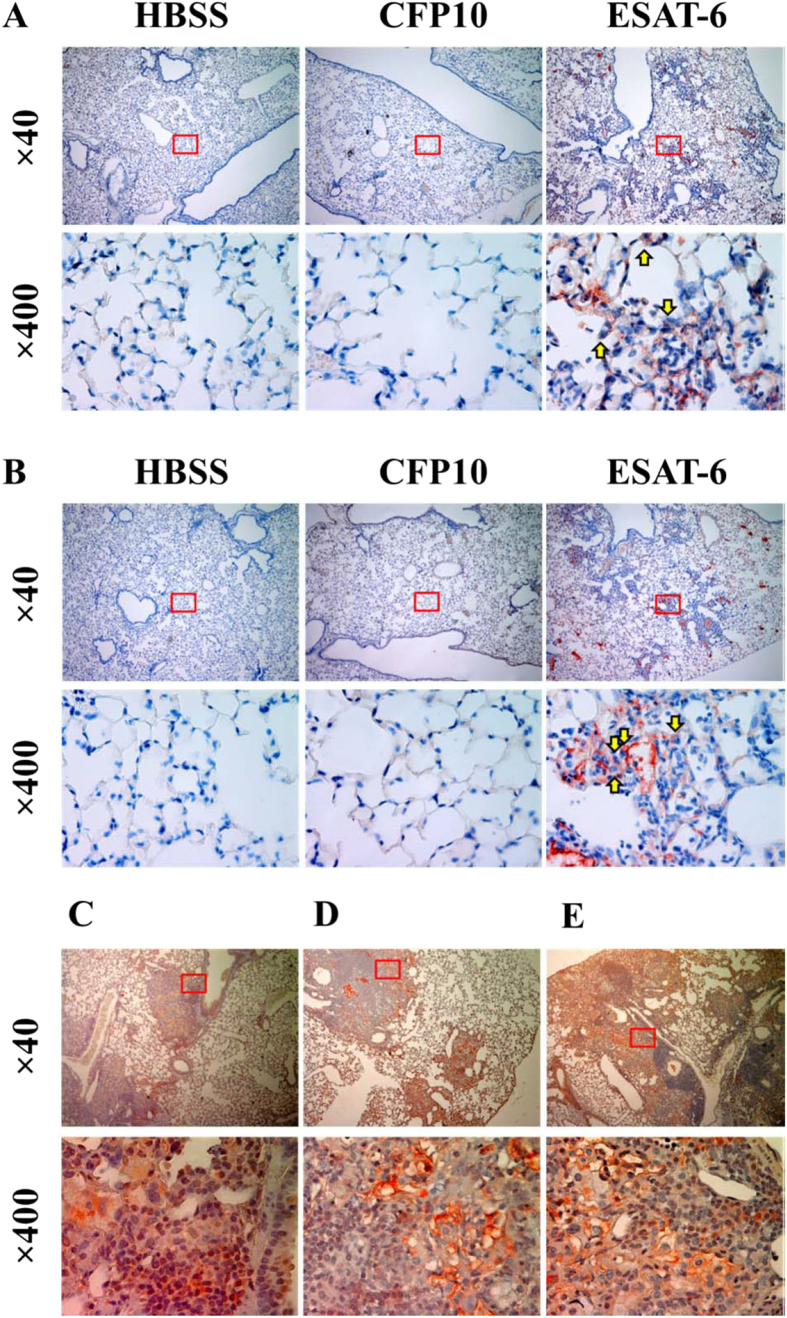
Administration of ESAT-6 but not CFP10 or infection of *Mtb* induces expression of ESAT-6, activation of STAT3 and production of IL-6 in the mouse lungs. C57BL/6 mice were given ESAT-6 or CFP10 at 40 μg/mouse or equal volume of HBSS (vehicle) intranasally. Three days after treatment, production of IL-6 (**A**) and phosphorylation of STAT3 (**B**) in the mouse lungs were examined by immunohistochemistry. One representative result from three mice is shown. Lung tissues from *M.tb* strain Erdman infected CBA/J mice were analyzed for expression of IL-6 (**C**), phospho-STAT3 (**D**) and ESAT-6 (**E**) after immunohistochemistry staining. One representative result from five mice is shown. For all panels, ×400 images are magnification of the insets in the corresponding ×40 images above.

To confirm our *in vitro* findings in live animals, we administered ESAT-6, CFP10 or HBSS (vehicle control) intranasally into the mouse for 3 days and evaluated the production of IL-6 and the activation of STAT3 in the mouse lungs by immunohistochemistry. Compared to CFP10 or HBSS, ESAT-6 induced infiltration and aggregation of inflammatory cells in the lungs consisting of mostly macrophages. ESAT-6, but not CFP-10 or HBSS, significantly induced IL-6 production (Fig. 8A) and STAT3 phosphorylation in mouse lungs (Fig. 8B), as indicated by red brown color staining, which was mostly located in the alveolar macrophages (Fig. 8B, arrow in lower panels). This indicated that ESAT-6 indeed induces activation of STAT3 and production of IL-6 *in vivo*. To confirm the expression of ESAT-6, production of IL-6 and activation of STAT3 in live *Mtb* infection, we examined these in the lung sections of mice that had been infected with *Mtb* Erdman for 120 days^39^ by immunohistochemistry. As reported by others^40^, the lungs of mice infected with *Mtb* had strong staining for ESAT-6 which was predominantly distributed in macrophages (Fig. 8E), suggesting active secretion of ESAT-6 by *Mtb* in the mouse lung macrophages. We also observed positive staining for IL-6 (Fig. 8C) which was distributed diffusely through large macrophage/lymphocyte aggregates that are typical of CBA/J mice after 120 days of *Mtb* infection. Phosphorylated STAT3 (Fig. 8D) was evident within and at the periphery of large macrophage/lymphocyte aggregates and also at high levels within small intra-lesional clusters of macrophages. Surrounding lung tissues were negative for IL-6, phosphorylated STAT3 and ESAT-6, suggesting that ESAT-6 production during live *Mtb* infection may also activate STAT3 to stimulate IL-6 production in the local granuloma. Of note, p-STAT3 was also observed in macrophage rich areas within granulomas from IL-10 KO mice (not shown).

## Discussion

Secretion of ESAT-6 by *Mtb* through ESX-1 secretion system plays a critical role in *Mtb* pathogenesis. Although ESAT-6 mediated cell damage and *Mtb* escape from phagolysosomes contribute to pathogenesis, modulation of host immune responses by ESAT-6 may provide additional mechanism for *Mtb* pathogenesis. In support of this, we showed that ESAT-6 stimulates IL-6 transcription and production by macrophages through activation of STAT3 which is independent of TLR2, IL-6 and IL-10 signaling. Both chemical inhibition and genetic silencing of STAT3 in macrophages inhibited ESAT-6-induced IL-6 production. This was supported by data from the macrophages infected with *Mtb* with *esat-6* deletion and intranasal administration of ESAT-6 into mouse lungs and immunohistochemical examination of *Mtb* infected mouse lungs for expression of ESAT-6, phospho-STAT3 and IL-6. Thus, this study describes a novel role for ESAT-6 in the initiation and amplification of inflammation at the early stages and in suppression of Th1 immune responses at the chronic phase^41^ of *Mtb* infection.

Understanding IL-6 production in TB infection is critical for deciphering TB pathology. Although IL-6 is required for early protective immunity against *Mtb* infection^42^,^43^, excess IL-6 production during chronic TB infection is associated with pathology. Because IL-6 levels in TB patients are associated with pathological changes and manifestation of clinical symptoms, such as thrombocytosis and C reactive protein response^44^, elevated serum IgG content^45^,^46^ and suppressed Th1 responses which is protective against TB infection^21^,^22^,^41^. The concentrations of serum IL-6 from TB patients are correlated with disease severity and changes in clinical parameters^44^ and spontaneous production of elevated IL-6 together with other inflammatory cytokines is also correlated with development of the lung lesions and their severity in patients with pulmonary TB^47^,^48^. Interestingly, compared to other inflammatory cytokines, the concentration of IL-6 together with IL-1β and IL-8 was the most abundant in the bronchial alveolar lavage of TB patients with extensive lung disease and correlated with disease severity as shown by high resolution computed tomography of affected lung areas of TB patients^48^. Infection of macrophages by *Mtb* stimulates IL-6 and other inflammatory cytokines and this has been attributed to cell wall components of *Mtb,* such as, muramyl dipeptide^49^ and lipoarabinomannan^50^. This may explain why BCG is capable of stimulating IL-6 production by macrophages^21^ despite its deficiency in ESAT-6 and its secretion machinery. In addition to cell wall components, the culture filtrate proteins of *Mtb* were also shown to induce IL-6 production by macrophages but without clear protein identity. This study adds ESAT-6 as another major component of *Mtb* that stimulates macrophage IL-6 production. This is supported by data from macrophages infected with *Mtb* H37Rv and its *esat-6* deletion mutant. Infection with H37Rv induced significantly higher amounts of IL-6 by both murine and human macrophages than its *esat-6* deletion mutant. Surprisingly we also noticed that CFP10 at higher concentration also induced macrophage IL-6 production though significantly less than that stimulated by ESAT-6. This is probably due to CFP10 interaction with macrophages, as we showed previously with human macrophages^7^. These together support the notion that ESAT-6 serves as an additional factor that contributes to *Mtb* infection induced IL-6 production by macrophages.

Trace amounts of contaminants may have been the contributing factors to ESAT-6 stimulation of cytokine production including IL-6 by macrophages. Although LPS is a potent IL-6 inducer, and contamination of trace amounts of LPS in ESAT-6 preparations may contribute to the production of IL-6 and other cytokines by macrophages as we have used recombinant proteins purified from the *E. coli* lysate. However, our results with polymyxin B (Fig. 1F) demonstrated that this is less likely. Although polymyxin B is less efficient in overall blocking of LPS, it has been shown to block efficiently LPS of *E. coli*^25^. Consistent with this, polymyxin B at10 μg/ml completely blocked IL-6 production by macrophages stimulated by 100 ng/ml LPS with an *E. coli* origin from Sigma. Contamination of muramyl dipeptide could not be an issue as these proteins were not purified from *Mtb* strains. Although peptidoglycan may be another contributing factor, it was not detectable in ESAT-6 by mass spectrometry analysis^10^. Hence our results clearly demonstrated that ESAT-6 stimulates IL-6 production by macrophages.

ESAT-6 is a major secreted protein of *Mtb* as *Mtb* is believed to reside in the membrane bound phagosomes or phagolysosomes of alveolar macrophages, one concern is the availability of free extracellular ESAT-6 for interaction with infected and non-infected bystander macrophages to stimulate STAT3 activation and IL-6 production. However, there is evidence which supports the fact that free ESAT-6 is available for interaction with immune cells during live *Mtb* infection. As B cells recognize intact protein antigens, recent studies reporting elevated titers of ESAT-6 antibodies in TB patient sera^51^,^52^, support the existence of free ESAT-6 in extracellular space of tissues during TB infection. Indeed it was shown recently that dendritic cells infected with *Mtb* release intact *Mtb* proteins including ESAT-6^53^. Furthermore, *Mtb* exists in the extracellular space during live infection in the lungs despite it is an intracellular pathogen^54^. Therefore, it is reasonable to speculate that free ESAT-6 exists in the extracellular space to interact with macrophages in both autocrine and paracrine manners.

Although we showed that ESAT-6 binds to immune cells including macrophages^7^, supportingdirect stimulation of macrophages by ESAT-6, the molecular identity of the ESAT-6 interacting components of immune cells remains unclear. Both TLR2 and TLR4 are the most dominant TLRs of macrophages in *Mtb* infection and play critical roles in macrophage activation and cytokine production. TLR2 was shown to directly bind to C-terminus of ESAT-6 and play a critical role in ESAT-6 regulation of immune responses^32^,^33^,^34^. However, our data with TLR2 knockout mouse macrophages did not support this and lack of TLR2 did not affect ESAT-6 induced STAT3 activation and IL-6 production by macrophages (Fig. 5A and B). Although we could not provide a clear rational for this discrepancy, the existence of other potential ESAT-6 binding proteins may be a contributing factor^55^,^56^, and requires further clarification. ESAT-6 activation of STAT3 in macrophages is a surprising finding as STAT family of transcription factors are activated by cytokines and growth factors and both IL-6 and IL-10 have been shown to be the major contributing factors to the activation of STAT3 in macrophages^35^. However, our studies with neutralizing both of these cytokines did not support their involvement in STAT3 activation by ESAT-6. Although activation of STAT3 in macrophages infected with *Mtb* was shown to be mediated by IL-10^36^, the activation of STAT3 was evident only at 3 h post infection later than that in our system even with *Mtb* infection, which is evident as early as one hour after infection and increased steadily until 16 h post infection (Fig. 6). This continued activation of STAT3 may be due to secondary effects of cytokines including both IL-10 and IL-6. The elevated STAT3 activation and IL-6 and IL-10 production by macrophages in a positive feedback mechanism may contribute to the pathogenesis of *Mtb* infection.

Regulation of IL-6 is controlled at the transcriptional level by several transcription factors through interaction with its regulatory elements. NF-κB plays a critical role in IL-6 transcription in addition to AP-1, NF-IL-6 and STAT3. STAT3 was shown to be a key signaling molecule in *Mtb* infected macrophages^28^,^29^ and macrophages with activated STAT3 in the peripheral blood of TB patients were shown to be pathogenic associated with TB progression^57^. Consistent with this, our Western blot results show that ESAT-6 induced STAT3 phosphorylation in macrophages (Fig. 2A), which is supported by confocal microscopy data indicating activation of STAT3 and its localization in both cytoplasm and nucleus of macrophages (Fig. 2B). This is consistent with a previous report showing both mitochondrial and nuclear localization of active STAT3 in the cells^31^. The EMSA data showed that STAT3 and NF-κB from macrophages stimulated with ESAT-6 bound to both the STAT3 consensus binding site and IL-6 proximal promoter, clearly demonstrating the functional significance of activated STAT3 by ESAT-6 (Fig. 2C–E). This was further supported by the data from STAT3 silencing and chemical inhibition (Figs 3 and 4). One interesting finding was that rapamycin, the inhibitor of mammalian target of rapamycin (mTOR), did not affect ESAT-6 stimulated IL-6 production despite it is shown to enhance LPS stimulated macrophage activation and inflammatory cytokine production by inhibiting IL-10^58^,^59^. This might suggest potential differences in the signaling pathways between ESAT-6 and LPS stimulated IL-6 production by macrophages and that the LPS stimulated IL-6 production by macrophages is sensitive to rapamycin. These together suggest that ESAT-6 stimulates IL-6 production by macrophages through activation of STAT3 as well as NF-κB.

Macrophage response by secretion of IL-6 is probably not specific to *Mtb* infection considering the pathological nature of over produced IL-6 in other chronic infectious diseases. This mechanism is also shared by several other pathogens^60^,^61^,^62^ including HIV^63^. HSP60 of *Helicobacter pylori*^64^ and gp120 of HIV have been shown to stimulate IL-6^27^. Therefore, activation of STAT3 and induction of IL-6 production may be a common mechanism shared by a variety of pathogenic microorganisms including *Mtb*. Activation of STAT3 and production of IL-6 by *Mtb* infected macrophages play significant role in host immune responses and pathophysiology of TB^29^, this study provides a novel evidence that ESAT-6, an essential virulence factor of *Mtb,* activates STAT3 and stimulates IL-6 production by macrophages. Considering the significance of the IL-6/STAT3 axis in different diseases, detailed signaling pathways of ESAT-6 mediated activation of STAT3 requires further study. Due to the *in vitro* nature of this study, the findings described here require further examination by animal models of *Mtb* infection to establish the biological relevance of ESAT-6 induced STAT3 activation and IL-6 production in TB infection.

In conclusion, we have identified a novel mechanism for *Mtb* infection induced IL-6 production by macrophages through ESAT-6 mediated activation of STAT3. Although detailed signaling pathways for such action of ESAT-6 in macrophages remain to be addressed, this may have significance in understanding the role of ESAT-6 in TB pathology.

## Materials and Methods

### Antibodies and chemicals

The antibodies for phospho-STAT3 at Y705, STAT3 (124H6), phospho-STAT1 at Y705, STAT1 and phospho-STAT5 at Y694 were from Cell Signaling Technology. Polyclonal rabbit anti-STAT3, anti-NF-κB p65 and isotype matched IgGs for supershift assays were from Santa Cruz Biotechnology. Horseradish peroxidase-conjugated goat anti-rabbit IgG and the goat anti-mouse IgG were from Bio-Rad and Santa Cruz Biotechnology, respectively. Antibodies against mouse IL-6, IL-6 receptor α (gp80) and IL-10 were from R&D systems. STAT3 inhibitors, Stattic and S3I-201 were from Santa Cruz Biotechnology. Rapamycin, Pam3CSK4 and lipopolysaccharide (LPS) were from Sigma.

### Mtb strains

Mtb H37Rv, its esat-6 deletion mutant (H37Rv::∆3875) or complemented strain (H37Rv::∆3875 C) were provided by Dr. David Sherman (University of Washington, Seattle, WA). The bacteria were cultured and prepared for infection as previously described^9^.

### Recombinant mycobacterial proteins

Recombinant ESAT-6 and CFP10 were expressed in *E. coli* and prepared as described earlier^7^,^9^. Recombinant plasmid containing Ag85A gene (Rv3804) was obtained from BEI resources and prepared as previously described^7^. Purity of Ag85A was >95% based on SDS-PAGE and Coomassie Brilliant Blue staining (not shown). The LPS content of recombinant proteins was below 39 pg/mg protein based on QCL-1000 Limulus amebocyte assay (Cambrex). Purified proteins were resuspended at 2 mg/ml in Hanks’ balanced salt solution (HBSS), aliquoted, and stored at −80 °C.

### Human monocyte-derived macrophages

Human CD14+ monocytes were purified from the peripheral blood mononuclear cells of 9 QuantiFERON-TB GOLD test negative healthy donors as described before^9^ after obtained signed consent forms from all the donors. All the experiments with human cells were performed following the NIH guidelines and regulations and the protocols approved by the Institutional Review Board of the University of Texas Health Science Center at Tyler. CD14+ cells were resuspended at 5 × 10^5^ cells/ml in RPMI-1640 medium (Life Technologies) supplemented with 25 ng/ml M-CSF (R&D Systems), 10% heat-inactivated human serum (Atlanta Biologicals), 100 u/ml penicillin, 100 μg/ml streptomycin, 1 mM sodium pyruvate and 0.1 mM MEM nonessential amino acids and were cultured in 24-well tissue culture plates at 2000 μl/well for 5 days to differentiate.

### Mouse bone marrow-derived macrophages

The animal studies were approved by the Animal Care and Use Committee of the University of Texas Health Science Center at Tyler and all the mice experiments were performed in accordance with the NIH guidelines and regulations. Pathogen-free female 6–8-week-old wild-type and TLR2 knockout C57BL/6 mice were purchased from the Jackson Laboratory. Mouse bone marrow-derived macrophages (BMDMs) were prepared as previously described^65^ with minor modifications. Briefly, bone marrow was flushed out from mouse femur and tibia. After red blood cell lyses, the cells were plated at 4 × 10^6^ cells in a bacteriological petri dish in 10 ml DMEM/F12 medium (Gibco) containing 10% fetal bovine serum (FBS), 100 u/ml penicillin and 100 μg/ml streptomycin, and supplemented with 20% (volume) L-929-conditional media. On day 3, another 5 ml of DMEM/F12 medium with 20% conditional media were added. After 7 days, the cells were harvested for further studies.

### Mouse alveolar macrophages

Broncho-alveolar lavage were collected from 6–8-week-old female C57BL/6 mice as described^65^ with minor modification. Briefly, an 18-gauge cannula inserted into the trachea and the lungs were washed with 1 ml PBS by flushing in and out for several times. The wash solutions were collected into a 15 ml conical tube and centrifuged at 600 × g for 8 min and the cells were resuspended in DMEM containing 10% FBS and 1% antibiotics for further studies.

### Mouse alveolar macrophage cell line

Mouse alveolar macrophage cell line RAW 264.7 was purchased from the American Type Culture Collection. The cells were maintained in DMEM containing 10% FBS with antibiotics at 37 °C, 5% CO_2_ until use.

### Determination of IL-6 concentration

IL-6 levels in the culture supernatants were determined by Human IL-6 ELISA MAXTM Standard or mouse IL-6 ELISA MAXTM Standard (both from Biolegend) following the manufacturer’s instructions.

### Preparation of total cell, nuclear and cytoplasmic protein extracts

Total cell proteins were prepared as previously described^7^. The nuclear and cytosolic protein extracts were prepared as previously described^66^. The concentrations of protein extracts were determined by a BCA assay (Thermo Scientific), aliquoted, and stored at −80 °C.

### Western blot

The proteins were separated by 10% SDS-PAGE and electro-blotted to nitrocellulose membrane in Tris-glycine buffer containing 20% methanol. The membrane was blocked with 5% skim milk in TBS for 1 h at room temperature and incubated with a primary antibody in TBS-T (TBS containing 0.05% Tween^®^ 20) with 5% BSA overnight at 4 °C. After washing with TBS-T, the membrane was incubated with horseradish peroxidase-conjugated secondary antibody diluted in TBS-T with 5% skim milk for 45 min at room temperature. After washing four times with TBS-T and once with TBS, antibody-bound proteins were detected by ECL (GE Healthcare).

### Electrophoretic Mobility Shift Assay

Electrophoretic mobility shift assay (EMSA) was performed as previously described^66^ by incubating 3–6 μg protein extracts with 32P-labeled DNA probes on ice for 30 min. The double-stranded oligonucleotides used as DNA probes were: STAT3, 5′-GATCCTTCTGGGAATTCCTAGATC-3′ (p27); mouse IL-6 proximal promoter (−75 to −55), 5′-TGGGATTTTCCCATGAGTCTC-3′ (28, 29); NF-kB, 5′-AGTTGAGGGGACTTTCCCAGGC-3′^66^; CREB, 5′-AGAGATTGCCTGACGTCAGAGAGCTAG-3′ (26).

### Real-time PCR

The expression of IL-6 mRNA in BMDMs was measured by Real-Time PCR from the total RNA of the cells using glyceraldehyde 3-phosphate dehydrogenase transcripts as internal control and calculating fold changes of IL-6 mRNA in the cells with treatment over that in cells without stimulation as previously described^7^.

### MTT assay

To determine cell viability, the MTT colorimetric assay was performed as previously described^7^.

### Transfection of siRNA

BMDMs with 50% confluence were transfected with either scrambled or STAT3 siRNA (Cell Signaling Technology) using Lipofectamine^®^ 2000 Transfection Reagent (Life Technologies) following the manufacturer’s instructions. The cells were harvested 48 h later, and plated in a 96-well plate at 2 × 10^5^ cells/ml in 200 μl per well DMEM/F12 with 10% FBS. The cells were incubated with or without ESAT-6 at 1 μg/ml for 24 h and IL-6 levels were determined by ELISA. For determination of silencing efficiency, some of the transfected cells were lysed in SDS-PAGE sample buffer, resolved by 10% SDS-PAGE, and transferred to a nitrocellulose membrane for blotting STAT3. The blot was then stripped and reblotted for STAT1 and GAPDH as specificity and loading controls, respectively.

### Immunofluorescence staining

Immunofluorescence staining was performed as previously described with minor modification^10^. Briefly, BMDMs adhered on sterile coverslips were incubated with 1 μg/ml ESAT-6 for 60 min, and then fixed with 1% paraformaldehyde in PBS on ice for 30 min. The cells were washed with PBS containing 5% FBS (staining buffer) and permeablized by incubating with 0.1% Triton X-100 in PBS for 15 min. After washed with staining buffer, the cells were incubated with mouse anti-phospho-STAT3-Y705 or isotype control mouse IgG for 2 h on ice. After washing, the cells were incubated with Alexa Fluo-568-conjugated goat anti-mouse IgG for 45 min. The cells were washed and incubated with DAPI. The coverslips were mounted on glass slides and the images were captured by Nickon Eclipse TE2000-5 inverted fluorescence microscope and analyzed with Ultra-View LCI scanning confocal system (PerkinElmer, Life Sciences).

### Immunohistochemistry

Female 6–8-week-old C57BL/6 mice were randomly divided into 3 groups consisting of 3 mice each. The mice were intranasally administered 40 μl of HBSS, 40 μg of CFP10 or ESAT-6 in HBSS^10^. Three days later the lungs were removed and instilled with 5% formalin in PBS for fixation. The fixed lungs were embedded in paraffin and sectioned and stained for phospho-STAT3 and IL-6. Lung sections from CBA/J mice that had been infected with *Mtb* strain Erdman (50–70 CFU) for 120 days^39^ were stained for ESAT-6, phospho-STAT3 and IL-6. For immunohistochemical staining, the lung sections were deparaffinized and rehydrated before antigen retrieval by incubation with sodium citrate buffer. After blocking by serial incubation with hydrogen peroxide, Ultra V block, and 5% goat serum in PBS, the lung sections were incubated with anti-mouse IL-6 mAb (1:25) or anti-phospho-STAT3 (1:100) overnight at 4 °C in humidified chamber. After incubated with biotinylated goat anti-mouse IgG and streptavidin-HRP, the sections were rinsed with PBS and incubated with 3-amino-9-ethylcarbazole, chromogenic HRP substrate, followed by counterstaining with hematoxylin. The images were captured using Ultra Vision Plus Detection System (Thermo Scientific).

### Statistical analysis

Data are expressed as mean ± standard error of the mean. Student’s t-test was performed for statistical analysis using GraphPad Prism version 4.0 software (GraphPad Software, Inc.). A P < 0.05 was considered statistically significant.

## Additional Information

**How to cite this article**: Jung, B.-G. *et al*. Early Secreted Antigenic Target of 6-kDa of *Mycobacterium tuberculosis* Stimulates IL-6 Production by Macrophages through Activation of STAT3. *Sci. Rep.* **7**, 40984; doi: 10.1038/srep40984 (2017).

**Publisher's note:** Springer Nature remains neutral with regard to jurisdictional claims in published maps and institutional affiliations.
